# Tracking the When, Where, and With Whom of Alcohol Use: Integrating Ecological Momentary Assessment and Geospatial Data to Examine Risk for Alcohol-Related Problems

**DOI:** 10.35946/arcr.v36.1.04

**Published:** 2014

**Authors:** Bridget Freisthler, Sharon Lipperman-Kreda, Melina Bersamin, Paul J. Gruenewald

**Affiliations:** Bridget Freisthler, Ph.D., is an associate professor at the UCLA Department of Social Welfare, Los Angeles, California. Sharon Lipperman-Kreda, Ph.D., is a research scientist; Melina Bersamin, Ph.D., is a research scientist; and Paul J. Gruenewald, Ph.D., is scientific director/senior research scientist, all at the Prevention Research Center, Pacific Institute for Research and Evaluation, Oakland, California.

**Keywords:** Alcohol use, abuse, and dependence, alcohol-related problems, problem drinking, alcohol use pattern, prevention, context, social context, risk factors, predictive factors, technology, electronic health technology, data collection and analysis, ecological momentary assessment (EMS), geospatial data, activity-space analysis

## Abstract

Prevention researchers have found that drinking in different contexts is related to different alcohol problems. Where and with whom people drink affects the types of alcohol-related problems they experience. Consequently, identifying those contexts that result in the greatest number of problems provides a novel opportunity to target new prevention efforts aimed at those contexts. However, identifying these contexts poses methodological challenges to prevention research. To overcome these challenges, researchers need tools that allow them to gather detailed information about when and where people choose to drink and how contextual factors influence drinking risks. New data collection and analysis techniques, such as activity-space analysis, which examines movement through different contexts, and ecological momentary assessment, which captures microlevel contextual changes as individuals move through their days, can advance the field of alcohol studies by providing detailed information on the use of drinking contexts, particularly when combined. Data acquired through these methods allow researchers to better identify those contexts where and conditions under which drinking and problems related to drinking occur. Use of these methods will allow prevention practitioners to target prevention efforts to those contexts that place most drinkers at risk and tailor prevention efforts to each context for specific outcomes.

The study of drinking contexts has become an increasingly fertile area of research in prevention science. Each drinking event has a series of contextual characteristics unique to that event. Where (location), when (the sequence of events), with whom (social characteristics), and under what circumstances (situation characteristics) a person drinks affect the types of alcohol-related consequences a person experiences. Taken together, these contextual characteristics may exacerbate or buffer an individual from experiencing alcohol-related problems. Through the identification of high-risk contexts, a selective prevention approach (Institute of Medicine and National Research Council 2009) can be applied by (1) targeting prevention efforts to contexts that place most drinkers at risk, and (2) tailoring prevention efforts to each context for related outcomes. However, for these efforts to be successful, the various characteristics of drinking contexts that initiate and reinforce drinking problems must be identified and understood.

Consider the following scenarios: After the senior prom, a girl goes to her boyfriend’s house for the night with a group of friends and their dates. The parents, who are hosting the party, set up the party but stay in their room to keep out of the way and let the teens enjoy themselves. The teens all think that the parents do not care if anyone drinks. Some already have consumed alcohol at the prom, and others invite along additional friends they know, some of whom are 21 years old. In another scenario, after the prom, another girl goes to the official “after party” hosted by the school at a local bowling alley. Because this party is hosted by the school, several parent chaperones are present. Some of these teens also have consumed alcohol at the prom, but they do not expect that they will be able to do so at the school-sponsored after party. In each scenario, the drinking context either provides specific risks for young people or buffers them from those risks. In the first example, the young people are at risk for drunkenness and other related problems, such as risky sex, arguments, and fights. In the second example, alcohol-related consequences are minimized, because contexts for both drinking and problems are constrained.

Recent research has found that different drinking contexts present unique risks for several social problems. Drinking at fraternity and sorority houses or events is related to more drinking-related problems among college students (Gruenewald and Ponicki 2009), including alcohol-related sexual intercourse (Bersamin et al. 2012). Parents who drink more frequently at bars, at home, or at parties use physical abuse more often, whereas parents who drink more often at restaurants use physical abuse less often (Freisthler and Gruenewald 2013). Drinking at bars is also related to a 12 percent increase in the likelihood of physically abusing a child, compared with only 1 percent for drinking at home or at parties (Freisthler and Gruenewald 2013). Males in relationships who drink more often at parties in other people’s homes seem to be more likely to commit intimate partner violence (IPV) (Mair et al. 2013) than their counterparts who drink less often at parties. Among young people, half of all episodes of intoxication occur in private homes as opposed to licensed premises or school events (Storvoll et al. 2010), and drinking and driving is more often when drinking occurs away from home (Tin et al. 2008; Walker et al. 2005). To tailor effective prevention strategies that respond effectively to contextual risk factors, it is necessary to gain insight into how location and temporal, social, and situation characteristics operate independently and conjointly to affect drinking and drinking-related outcomes. Ultimately, this information may result in police increasing patrols of risky drinking contexts (e.g., parking lots during a football game); parents being educated about high-risk settings; and specialized prevention materials, resources, and services provided at relevant locations.

This article presents a social ecological model that explicates relationships among key individual and contextual factors involved in alcohol use and alcohol-related problems. This is followed by a discussion of two complementary approaches to obtain the detailed information necessary to examine these relationships: activity-space analysis (ASA) and ecological momentary assessment (EMA). ASA highlights the importance of examining movement through different contexts and its resulting impact on drinking behaviors and related problems. EMA is a methodological tool that captures microlevel contextual changes as individuals move through their days. Integration of these two approaches in studying risks for alcohol-related problems can (1) provide a framework for identifying the key contextual elements that place drinkers at risk, and (2) allow researchers to examine linkages in time among behaviors, contextual elements, and where people regularly spend time.

## A Social Ecological Framework of Drinking Contexts and Alcohol-Related Problems

**Figure 1:**
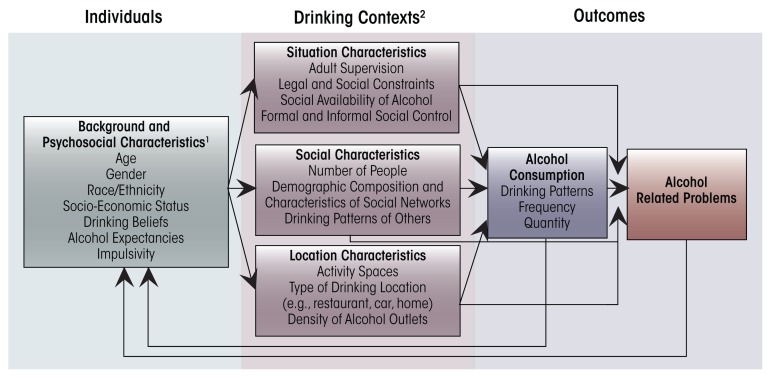
Social–ecological framework of drinking contexts and alcohol-related problems. NOTES: ^1^ The characteristics listed in each box are provided as an example. They are not an exhaustive list of variables one might include in social–ecological models. ^2^ Macro- (e.g., alcohol outlet density) and micro-level drinking contexts (e.g., drinking location) are included in the model above, but we focus primarily on micro-level drinking contexts here.

Alcohol-related problems are affected by individual characteristics; the situational, social, and locational characteristics of drinking contexts; and alcohol use. Figure 1 presents a conceptual outline of relationships among these social ecological aspects of drinking environments. Relationships among components of the figure reflect some of the social mechanisms by which characteristics of drinking contexts may affect alcohol use and related problems among drinkers. These mechanisms refer to the causal processes by which interactions among different factors in individuals’ macro-ecological environment (e.g., alcohol outlets and type of drinking location) affect the social behaviors of individuals (Gruenewald et al. 2014). Drinking contexts may modify how much alcohol a person consumes, affecting the likelihood of that person experiencing alcohol-related problems (as seen by the lines connecting drinking contexts to alcohol consumption). These contexts also may increase risks for people who are in them, regardless of their own drinking behavior (as evidenced by the lines connecting aspects of drinking context directly to alcohol-related problems). For example, a person who spends a lot of time in bars, even if he or she does not drink, might be more likely to be the victim of an assault.

Individual characteristics refer to demographic and psychological factors such as impulse control, self-efficacy, risk-taking propensity, and drinking expectancies. The selection of different drinking environments seems to be correlated with a variety of different personal characteristics such as gender, age, and ethnicity and personal characteristics such as impulsivity, risk taking, drinking expectancies, and beliefs (Gruenewald et al. 2014). In the previous example of the high-school prom, personal characteristics such as risk taking or poor impulse control may lead a student to choose to attend the house party instead of the school-sponsored party at the bowling alley. Once in a drinking context, that context may have an associated set of norms and rules that dictate the appropriate amount of drinking and the related behaviors (e.g., aggression), which also may influence consumption and alcohol-related problems in that setting (Demers et al. 2002; Kairouz et al. 2002). As shown in figure 1, prior alcohol consumption and alcohol problems in a specific context may affect an individual’s future drinking behaviors and beliefs. For example, if the high-school student felt more social after drinking alcohol at the house party and did not experience any negative outcomes such as feeling sick or getting into trouble with his or her parents, he or she will have more positive expectations and will be more likely to drink alcohol at the next drinking event.

Situational characteristics refer to those features of a context that change from one drinking event to another, such as time of the day, level of parental supervision, or legal and social constraints. For example, it may be that specific locations place constraints on behavior such that some alcohol-related problems (e.g., aggressive behavior) may be less likely to occur at someone else’s home (as a result of social constraints) or restaurants and bars (as a result of legal constraints) but more likely to happen at less supervised locations such as parks and beaches. People who drink often at bars may be concerned about potential legal ramifications of fighting in bars and may be more likely to become aggressive at home (toward an intimate partner or children), where fewer individuals are present to stop the abuse (Cunradi 2010; Freisthler and Gruenewald 2013; Freisthler and Holmes 2012).

Social characteristics refer to the collective attributes of people and their relationships in drinking contexts. These characteristics also are temporal and change from one drinking event to another. An intimate house party with friends and significant others has different risks than a large party at a bowling alley where a person may or may not know all the other attendees. Social characteristics of the drinking context, such as number of people, gender or age composition, peer expectations, and drinking behaviors of social-network members also may be important determinants of alcohol-related consequences. Male college students reported greater frequency of drunkenness in large groups of mixed-gender and small groups of same-gender individuals compared with small mixed-gender groups (Senchak et al. 1998). Parents who receive social support resources in the form of social companionship (i.e., spending time with family or friends doing leisure activities) from individuals living in their neighborhood engage in physical abuse more frequently than parents who have lower levels of social support (Freisthler et al. 2014). Parents who regularly socialize with other parents may share discipline strategies, including forms of physical discipline that include corporal punishment or physical abuse. If these parents either respond positively to the use of these practices or do not respond at all, this may create a norm where use of physical abuse is informally sanctioned (Emery et al. 2014). Moreover, the presence of social companionship support in an area was moderated by alcohol outlet density, such that parents who lived in areas with higher density of on-premise outlets (i.e., bars) and a high level of social support used physical abuse more often compared with parents with high social support and low outlet density (Freisthler et al. 2014).

Returning to the prom example, a high-school freshman female who attends a party at which alcohol is served may have little experience with alcohol and may feel awkward because the majority of prom-goers are juniors and seniors, but she decides to drink at the after party to fit in with the rest of the group. The number of people drinking at the after party (a social characteristic) may place her at higher risk for alcohol-related consequences (e.g., hangover, sexual assault) than some of her peers who did not attend that party. Importantly, alcohol may not be a necessary ingredient for some problems, because this setting may draw together people with intentions to engage in risky behaviors, regardless of the amount they consume. Therefore, distinguishing the roles of alcohol in these social processes in drinking contexts is important.

Location characteristics, such as proximity to sources of alcohol or the necessity of using a motor vehicle to get from one place to another, will further affect the likelihood that one will experience negative outcomes. Within these contexts, alcohol use affects the rates at which problems will occur. Drinking in places with higher densities of alcohol outlets may increase bar hopping (i.e., drinking at multiple locations in succession such as at home, then at a restaurant during dinner, and finally at a bar for a nightcap). In addition, the availability of drinking places outside the home almost invariably leads to greater exposures to risks related to driving after drinking (Gruenewald et al. 2014). These risks may be compounded among individuals whose daily living activities bring them into regular contact with these places (e.g., higher outlet densities, closer to friends who drink heavily).

### Relationships Between Contextual Characteristics

Individual, situational, social, and locational aspects of drinking environments are temporal and change over the course of a day or evening. To further understand the social mechanisms by which drinking contexts affect alcohol use and drinking-related problems, it is important to assess the relationships among these different aspects of drinking contexts, drinking behaviors, and drinking-related outcomes over time. Thus, researchers need to collect detailed data that identifies where people spend time on a regular basis, whether alcohol is consumed in those settings, and with whom respondents are spending time. For example, a high-school student may start his or her day at home, leave for school, go to a part-time job, head over to a friend’s house to study, and end the day back at home. While at his or her friend’s house, he or she may find the liquor cabinet open and available for use. A parent also might start his or her day at home, leave to take the children to school, drop off clothes at the dry cleaner before heading into work, walk to a lunch meeting near the office, stop at the grocery store for some dinner items on the way home, pick up the children from an after-school program, and take them to activities before heading back home for the night. While at lunch the parent may find time to have a drink with a friend. In each of these different places, an individual’s risk for alcohol use or related problems will be a function of effects related to physical access to alcohol (i.e., proximity to sources of alcohol), the social interactions that may occur within places (i.e., lunch with a colleague), and individual proclivities to drink.

## Studying the Geography of Drinking Contexts and Related-Problems

As suggested here, location characteristics such as proximity to alcohol outlets are only one of several key components for understanding the roles of drinking contexts in drinking and related problems. Historically, these characteristics have been measured using standard geospatial approaches. Such approaches most often include the use of spatial data in Geographic Information Systems to map locations of alcohol outlets relative to locations where people live and presumably spend much of their time. Thus, almost all previous and current research on the physical availability of alcohol through outlets and alcohol-related problems has focused on local residential and commercial environments using administrative areas such as Census tracts or ZIP Codes (Freisthler et al. 2007; Gruenewald and Remer 2006). Such studies, however, do not account for the variations in exposures to drinking environments and opportunities for drinking that individuals encounter as they move through their days (Inagami et al. 2007).

Understanding the geographic distribution of “activity spaces” requires individual respondents to provide geographic information on where they regularly spend their time, at minimum, or a daily listing of where they go during each day, including the routes the individual has traveled to get to those places, at maximum. Activity spaces consist of the places that an individual has visited and the routes and areas the individual has traveled throughout each day. The way individuals use their environments, represented by their activity spaces, and social relationships within these spaces may mitigate or exacerbate drinking and related problems. Assessments of the structures of these spaces may provide better measures of exposures to alcohol use and related harms. The examination of activity spaces also can help explicate how exposure to an alcohol environment may differ between drinkers and nondrinkers, affecting their level of risk. Research has not determined (1) whether greater physical access to alcohol increases the risk for alcohol-related problems (e.g., assaults) for both drinkers and nondrinkers, and (2) the social mechanisms that may explain why differences may or may not exist.

**Figure 2:**
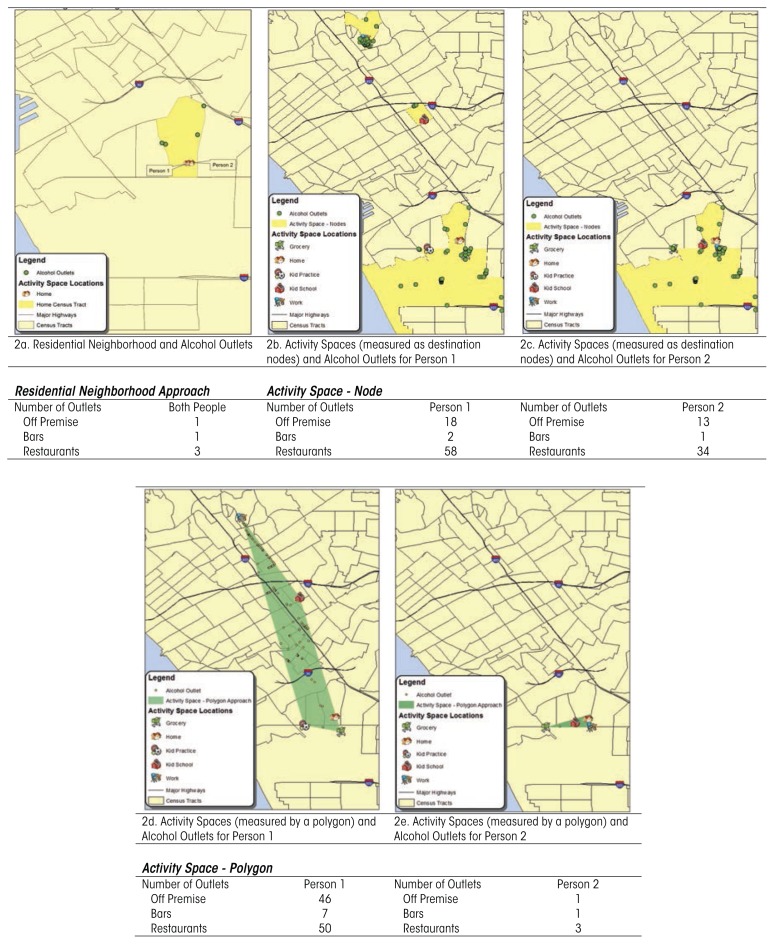
Depiction of physical availability of alcohol for two individuals using three different descriptions of a person’s environment.

Figure 2 shows how depicting the risks associated with the physical availability of alcohol differ when different definitions are used. Figure 2A measures the density of alcohol outlets within a residential neighborhood shared by two persons. This has been the practice, to date, of most neighborhood studies examining alcohol outlet density. Figures 2B and 2C show destination “nodes” of activity-space locations for the same two people. These nodes are the Census tracts surrounding the destination locations (e.g., grocery store) in each person’s activity space. These nodes also could be created using a buffer distance (e.g., 0.5 mile) around each of the points. Finally, figures 2D and E show polygons depicting the entire area covered by a person by connecting the locations in the activity space using a straight line. Alternatively, images could have been created using travel routes or putting buffer distances around those straight line or travel routes. Figure 2 shows how these various depictions result in different estimations of exposures to alcohol outlets for two people living in the same residential neighborhood. In this example, Person 1, with a larger activity space, has much more exposure to the physical availability of alcohol (78 outlets using the nodes approach, 103 outlets using the polygon approach) than he or she would if only the neighborhood alcohol outlets were considered (4 outlets). In this example, the activity space for Person 1 is much larger than that of Person 2, and the physical availability of alcohol is similarly expanded. Using residential neighborhood as a measure of exposure obscures the effect of exposure at the location where a person works 40 hours a week. Solely focusing on the exposures to alcohol and related-problems in a person’s residential neighborhood does not capture the full range of risk exposure for most individuals (Inagami et al. 2007).

### Tracking Activity: Collecting Place–Time Data

Researchers need reliable methods to collect specific geographic information that links the social mechanisms under study to the theoretical statements that tie behaviors to specific geographic locations. For example, an analysis of the mechanisms by which different types of social support (e.g., tangible vs. companionship), drinking behaviors of network members, and use of on-premise drinking places interact to increase risks for different forms of child neglect (e.g., leaving a child home alone without supervision) would require identification of locations at which adults were present when these events take place (e.g., a bar or house party) (Freisthler and Holmes 2012). However, explaining social mechanisms relating alcohol use to the risk of alcohol-related traffic crashes may require specific location information on the routes an individual travels to and from a drinking event, since risks for this outcome may increase by the complexity of roadways traveled (e.g., on and off ramps for highways, curving roads). Here, GPS devices that provide location data every second may be ideal (Rainham et al. 2010).

The few studies that have measured activity spaces have done so with primarily school-aged youth and small sample sizes and in isolated geographic areas (e.g., one city), limiting generalizability (Mason et al. 2009; Wiebe et al. 2013). These studies have used various approaches to elicit information about activity spaces, including “free listing,” in which participants were asked to list all of the places they went in the past week (Mason 2010); a “recall method,” in which participants reported their activities sequentially (e.g., “Where did you go after you woke up?,” etc.) (Mason 2010; Wiebe et al. 2013); and asking for locations of cross streets of places a person goes to regularly (Martinez et al. 2014). Researchers can then map these locations and connect the locations via polygons as in Figure 2 (Martinez et al. 2014) or create specific maps of individuals’ exact travel routes and their locations (Wiebe et al. 2013) for analysis. All three approaches elicit usable activity-space information for approximately 90 percent of all respondents. This suggests that a variety of approaches used to produce information on activity spaces of study participants can be successful. Although there is certainly promise in using these self-report approaches to capture activity spaces and therefore elucidate mechanisms of alcohol use and related problems, these techniques have not been well validated.

Challenges do exist when collecting these types of data. Activity-space data require that respondents provide locations that can be geocoded to report those points. Respondents may not always accurately report those locations. For example, when asked to pinpoint a location using names of two streets that intersect, they may name two parallel streets or identify two streets that do not intersect. This can be overcome by using GPS technology to track individuals’ movements across space. However, use of GPS often results in large amounts of data for each individual, making it difficult to analyze (Rainham et al. 2010). Finally, it is unclear how best to construct and analyze meaningful activity spaces that explore the full range of movements and exposures to harmful environments.

Recent research on substance abuse has combined activity space approaches with GPS devices to document micro-movements across space with information elicited at regular intervals from the respondent via EMA (Epstein et al. 2014). This activity-space information, along with additional geo-located data on alcohol-related problems, alcohol outlets, drinking events, and social-network members can be analyzed to understand how and where a person spends time, who else is likely to be in that environment, and how access to alcohol might interact resulting in a greater likelihood of experiencing alcohol-related problems.

In addition to specific geographic risks within an individual’s activity space, that space may include certain types of social contacts that may either buffer him or her from risks associated with the physical availability of alcohol or exacerbate those risks. A parent who spends time with impulsive social-support members at bars where violence occurs regularly may internalize those norms, placing his or her children at greater risk of being physically abused (Freisthler and Holmes 2012). These types of relationships currently are being combined with activity spaces of parents to identify the places where parents spend time, have easier access to formal sources of alcohol (e.g., bars, liquor stores), and have social supports that may increase the risk for child physical abuse and neglect. A similar mechanism may be at play if instead of a parent, the bar-goer is a spouse, elevating the risk for IPV (Cunradi 2010). Geospatial data alone do not provide information on who those contacts are, how they modify risks for alcohol-related problems, or how they explicate the mechanisms that may be susceptible to prevention efforts.

## Real-Time Assessment of Drinking Contexts and Alcohol-Related Problems

Activity-space analysis represents a step beyond the standard use of geospatial data to identify exposures to drinking contexts, opportunities to drink in those contexts, and problems related to those contexts. However, by themselves, activity-space analyses do not provide information about specific characteristics of drinking events that may be relevant to the further elucidation of the social processes that lead from opportunities to drink to drinking risks. Situation and social characteristics (see figure 1) may be better identified through EMAs.

EMA refers to a range of methods that involve collection of real-time data that can be used to describe a person’s behaviors and experiences throughout the day in his or her natural environments (Shiffman et al. 2008). Momentary assessments may target specific events of interest, such as drinking events, to study individuals’ behaviors and experiences related to these events in more detail and in relation to other pre-and postevents. Revisiting the prom example, whereas activity-space analysis forces us to think about all the different spaces that an adolescent might occupy on prom night (e.g., own home, dance, 25 miles of freeway, friend’s home) and exposure to alcohol in these spaces more broadly, EMA conducted at regular intervals over the course of the night could capture the predrinking event in the car before the dance; the transition from the dance location to the home location where the after-prom party occurred; access to alcohol at that particular home; drinking behaviors at the after-prom party; characteristics of those at the party (age and gender composition, adult supervision); and any alcohol-related problems experienced as a result of drinking during the evening, such as risky sex behavior.

A few limitations and challenges of the EMA approach should be considered (Shiffman et al. 2008). First, EMA reports are subject to error resulting from potential poor compliance. For example, it is possible that lack of participant retention in the EMA assessments, conducted during the prom night, may exclude important drinking events or characteristics. However, incentive methods such as offering participants a bonus for completing all assessments or delivering immediate incentives for each assessment completed by participants can be used to increase retention in EMA (Wiebe et al. 2011). Second, EMA methods collect self-reported data and therefore might be influenced by personal characteristics or biases, such as social desirability. Finally, EMA studies produce many observations ordered in time. It is necessary to maintain and consider these temporal data, especially when integrating EMA and activity-space data as described above.

Despite these potential limitations, there is great value to using EMA to study the role of drinking contexts in alcohol-related problems. First, EMA may obtain a more accurate report of behaviors and experiences in people’s natural environments. Traditional survey methods use predefined categories of drinking locations (e.g., party, restaurants/bars, home) and contextual characteristics that fail to provide an accurate picture of the wide range of drinking contexts that people encounter. Also, when asked about the past year, past month, or even past 24 hours, people may not accurately recall locations, setting characteristics, and drinking behaviors that were specific to a particular event. EMA attenuates recall errors and provides rich and descriptive event-specific information (Shiffman et al. 2008). The event-specific information that EMA provides allows researchers to identify and distinguish the contextual characteristics associated with specific alcohol-related behaviors to explain the social process by which these problems occur. Therefore, the use of EMA allows researchers to describe and test social mechanisms that explain why and how some drinking contexts result in more alcohol-related consequences than others.

Human behaviors change over time and across situations. Further, people’s experiences, choices, and behaviors in one situation are likely to affect their experiences, choices, and behaviors in another situation. For example, it is possible that supportive characteristics for aggression in the first drinking event (e.g., increasingly crowded bar, new drinking venue with a majority of male patrons) will contribute to the likelihood of engaging in physical aggression over the course of an evening. Therefore, such behavior may only manifest in the second or third drinking context with fewer legal ramifications. EMA data are necessary for understanding individuals’ experiences, choices, and behaviors over time and across contexts to establish knowledge about the potential dynamics by which contextual characteristics affects alcohol consumption and drinking-related problems.

Previous studies have used EMA approaches to study alcohol craving, use, dependence, and other drinking problems among youths, young adults, and adults (e.g., Collins et al. 2003; Kashdan et al. 2010; Todd et al. 2009). These studies highlight the importance of examining the episodic-and context-based nature of alcohol and other substance use. Clearly, to gain insight into how drinking contexts affect alcohol use and alcohol-related problems, it is necessary to measure what aspects of the environment fluctuate and in what order; hence the value of EMA. Less research, however, has used EMA approaches to investigate the role of drinking contexts in alcohol-related problems. Such research will inform prevention theory and guide prevention efforts in targeting high-risk drinking contexts.

A common EMA approach to studying drug use behaviors asks respondents to initiate assessments when they are involved in the alcohol or other drug (AOD) use behavior (i.e., event-based assessments) (Piasecki et al. 2001). They also are prompted at random times to complete similar assessments when not using AODs. Alternatively, study participants are prompted to respond to assessments at regular intervals or random times (Shiffman 2009). A recent study used text-prompted surveys to investigate young adults’ drinking behaviors before going to licensed premises, their alcohol consumption, and their drinking problems (Labhart et al. 2013). EMA with adults may include interactive voice-response surveys, whereas text-prompted surveys may be more appropriate for youth and young adults, given each population’s experience and familiarity with the technology. Choosing the most suitable EMA approach and the appropriate method depends on the research question as well as characteristics of the participants, such as age and access to smartphones or the Internet.

In the authors’ ongoing project, EMA data about drinking contexts, drinking behaviors, and related problems will be collected from 16-to 19-year-olds using cell phone interactive voice-response (IVR) surveys to assess the characteristics related to greater problems among youth. No GPS tracking data will be collected in conjunction with these EMA reports. Assessments will be collected at six time points (8 p.m. and 11 p.m. Friday; 11 a.m., 8 p.m., and 11 p.m. Saturday; and 11 a.m. Sunday) on 2 weekends over 2 consecutive weeks. Each time, participants will receive invitation calls on their cell phones to respond to a brief survey. In conjunction with survey data of youth individual characteristics (e.g., demographics and drinking beliefs), these EMA data will be used to address important questions related to where, when, and under what conditions youth experience specific problems.

In sum, the episodic-and context-based nature of AOD use makes the EMA approach appropriate for studying such behaviors (Shiffman 2009). EMA methods allow researchers to overcome a few limitations of traditional survey methods and study people in their natural environments. In addition, EMA methods allow researchers to examine in detail the contextual characteristics that may contribute to different human behaviors and, more importantly, how these characteristics and behaviors interact over time. Focusing on drinking contexts and alcohol-related problems, EMA data can examine type of drinking locations and how contextual factors uniquely or jointly contribute to drinking-related problems over time. Specifically, the microfocus and contextual and temporal nature of EMA data offer an opportunity to study important questions related to whether, to what extent, and how certain contexts or series of contexts amplify the risks associated with drinking.

## Conclusions

Each drinking event has a set of characteristics unique to that event. Previous research has shown that these characteristics affect the types of alcohol-related consequences that individuals experience. However, to design focused environmental prevention programs (i.e., changing the environment where drinking occurs) (Treno and Lee 2002) aimed at reducing these consequences in the contexts where they occur, more information about the mechanisms that link situations, people, and places are needed. More specifically, if we are able to identify key drinking locations, situations, and social characteristics that contribute to specific alcohol-related problems, more fine-tuned selective prevention programs that target aspects of drinking environments leading to those specific problems can be developed. These interventions may be tangentially related to alcohol use itself but are nevertheless quite effective. For example, crisis nurseries, which are intended for situations in which parents can no longer effectively supervise and care for their children (Cole and Hernandez 2011), could be used in situations in which addicted or dependent parents find they are no longer able to manage problems related to alcohol use. Such care may ensure the children remain safe during and after risky drinking events and reduce the incidence of child physical abuse and supervisory neglect. To reduce not only child abuse but prevent an increase of drinking and driving with children in the car, requirements might include that parents must either agree to leave the child overnight and/or that the person picking up the child must have a blood alcohol content (BAC) lower than 0.05 g/dL (Sen 2006). Informal versions of this intervention-like neighborhood babysitting co-operatives could provide sources of support for families with young children and be used even during evening hours, when parents may visit drinking venues or attend parties where drinking is likely to occur (Freisthler and Holmes 2012). Complementarily, alcohol outlets that offer child care services in combination with alcohol service to adults could be discouraged from doing so if they ultimately place children at greater risks of neglect and abuse subsequent to drinking events.

An example of prevention programs that make use of the contextual information targeting adolescent drinking would be informational apps for parents in which they can identify risky contexts. For example, an app could ask parents to obtain and provide information such as the location of the party (e.g., outdoor, home) their child wanted to attend, potential supervision, number of guests, and the likelihood of legal drinkers in attendance. The app would help parents identify the kinds of parties that are most dangerous to teens and offer guidance to reduce risks related to underage drinking, including guidance on local social host laws, actions to contact social hosts (unwitting or otherwise), and access to police and social service agencies. Part of the success of such an intervention might be the experience of going through a checklist that reminds parents to have conversations with their children about drinking risks and helps them explore their concerns with their children. Another app, similar to the LiveSafe app being implemented on college campuses, might encourage underage youth to report instances of drinking and related problems via text messages to the police. This information would allow police to intervene more quickly in contexts where risky drinking or alcohol-related problems have occurred. Perhaps more importantly, the app provides encouragement to youth to recognize and respond to risks in their drinking environments.

The social ecological framework presented here is a starting point to identifying aspects of the drinking environment that expose individuals to increased risk for alcohol-related problems. Activity-space analysis and EMA are the most suitable approaches to study these aspects and examine the mechanisms by which risk may occur. Individually, both of these approaches can test various aspects of the social ecological framework presented. However, integration of both provides a richer understanding of the underlying contexts that result in problems. Epstein and colleagues (2014) integrated activity spaces and EMA data to assess mood, stress, and drug craving among opioid-dependent polydrug users receiving methadone maintenance in the context of their environment. The authors referred to this approach as geographical momentary assessment. To the best of our knowledge, no other published study has integrated these methods.

Additional challenges may exist when integrating these types of data. First, training of study participants on the protocol, the assessments, and any device used is an essential aspect of such studies. Second, ethical considerations must be addressed when tracking an individual’s location and associated behaviors, because the protocols are detailed, data intensive, and potentially may intrude on other protected behaviors (Arora et al., in this issue). Third, the use of GPS technology and the EMA approach often result in large amounts of observation data for each individual, making it important to develop appropriate analysis strategies to answer key research questions (Rainham et al. 2010; Shiffman et al. 2008). Finally, as noted before, it is necessary to maintain and consider time data to link EMA assessments and GPS data and employ an appropriate analytical approach to analyze integrated data.

Despite these challenges, we believe that the integration of EMA and GPS data offers a great opportunity to overcome limitations of current research and enhance our understanding of the social mechanisms that underlie associations between drinking contexts and drinking-related problems. Revisiting the prom example, data from both activity-space analysis and EMA can help in theorizing how individual characteristics (e.g., risk taking), contextual characteristics (e.g., lack of adult supervision, other drinkers, gender composition), locational characteristics (e.g., home), pre-and postevents, and alcohol consumption (drinking before the event) interact to result in problem behaviors. Further understanding of such mechanisms has the potential to advance prevention theory, current research, and context-based interventions to prevent alcohol-related problems.

## References

[b1-arcr-36-1-29] Bersamin MM, Paschall MJ, Saltz RF, Zamboanga BL (2012). Young adults and casual sex: The relevance of college drinking settings. Journal of Sex Research.

[b2-arcr-36-1-29] Cole SA, Hernandez PM (2011). Crisis nusery effects on child placement after foster care. Children and Youth Services Review.

[b3-arcr-36-1-29] Collins RL, Kashdan TB, Gollnisch G (2003). The feasibility of using cellular phones to collect ecological momentary assessment data: Application to alcohol consumption. Experimental and Clinical Psychopharmacology.

[b4-arcr-36-1-29] Cunradi CB (2010). Neighborhoods, alcohol outlets and intimate partner violence: Addressing research gaps in explanatory mechanisms. International Journal of Environmental Research and Public Health.

[b5-arcr-36-1-29] Demers A, Kairouz S, Adlaf EM (2002). Multilevel analysis of situational drinking among Canadian undergraduates. Social Science & Medicine.

[b6-arcr-36-1-29] Emery CR, Nguyen HT, Kim J (2014). Understanding child maltreatment in Hanoi: Intimate partner violence, low self-control, and social and child care support. Journal of Interpersonal Violence.

[b7-arcr-36-1-29] Epstein DH, Tyburski M, Craig IM (2014). Real-time tracking of neighborhood surroundings and mood in urban drug misusers: Application of a new method to study behavior in its geographical context. Drug and Alcohol Dependence.

[b8-arcr-36-1-29] Freisthler B, Holmes MR (2012). Explicating the social mechanisms linking alcohol use behaviors and ecology to child maltreatment. Journal of Sociology and Social Welfare.

[b9-arcr-36-1-29] Freisthler B, Gruenewald PJ (2013). Where the individual meets the ecological: A study of parent drinking patterns, alcohol outlets, and child physical abuse. Alcoholism: Clinical and Experimental Research.

[b10-arcr-36-1-29] Freisthler B, Gruenewald PJ, Remer LG (2007). Exploring the spatial dynamics of alcohol outlets and Child Protective Services referrals, substantiations, and foster care entries. Child Maltreatment.

[b11-arcr-36-1-29] Freisthler B, Holmes MR, Price Wolf J (2014). The dark side of social support: Understanding the role of social support, drinking behaviors and alcohol outlets on child physical abuse. Child Abuse & Neglect.

[b12-arcr-36-1-29] Gruenewald PJ, Ponicki WR A Simple Mathematical Model of Drinking Patterns and Drinking Contexts.

[b13-arcr-36-1-29] Gruenewald PJ, Remer L (2006). Changes in outlet densities affect violence rates. Alcoholism: Clinical and Experimental Research.

[b14-arcr-36-1-29] Gruenewald PJ, Remer LG, LaScala EA (2014). Testing a social ecological model of alcohol use: The California 50-City Study. Addiction.

[b15-arcr-36-1-29] Inagami S, Cohen DA, Finch BK (2007). Non-residential neighborhood exposures suppress neighborhood effects on self-rated health. Social Science & Medicine.

[b16-arcr-36-1-29] Institute of Medicine and National Research Council (2009). Preventing Mental, Emotional, and Behavioral Disorders Among Young People: Progress and Possibilities.

[b17-arcr-36-1-29] Kairouz S, Gliksman L, Demers A, Adlaf EM (2002). For all these reasons I do drink: A multilevel analysis of contextual reasons for drinking among Canadian undergraduates. Journal of Studies on Alcohol.

[b18-arcr-36-1-29] Kashdan TB, Ferssizidis P, Collins RL, Muraven M (2010). Emotion differentiation as resilience against excessive alcohol use: An ecological momentary assessment in underage social drinkers. Psychological Science.

[b19-arcr-36-1-29] Labhart F, Graham K, Wells S, Kuntsche E (2013). Drinking before going to licensed premises: An event-level analysis of predrinking, alcohol consumption, and adverse outcomes. Alcoholism: Clinical and Experimental Research.

[b20-arcr-36-1-29] Mair C, Cunradi CB, Gruenewald PJ (2013). Drinking context-specific associations between intimate partner violence and frequency and volume of alcohol consumption. Addiction.

[b21-arcr-36-1-29] Martinez AN, Lorvick J, Kral AH (2014). Activity spaces among injection drug users in San Francisco. International Journal of Drug Policy.

[b22-arcr-36-1-29] Mason MJ (2010). Attributing activity space as risky and safe: The social dimension to the meaning of place for urban adolescents. Health & Place.

[b23-arcr-36-1-29] Mason MJ, Mennis J, Coatsworth JD (2009). The relationship of place to substance use and perceptions of risk and safety in urban adolescents. Journal of Environmental Psychology.

[b24-arcr-36-1-29] Piasecki TM, Jahng S, Wood PK (2011). The subjective effects of alcohol-tobacco co-use: An ecological momentary assessment investigation. Journal of Abnormal Psychology.

[b25-arcr-36-1-29] Rainham D, McDowell I, Krewski D, Sawada M (2010). Conceptualizing the healthscape: Contributions of time geography, location technologies and spatial ecology to place and health research. Social Science & Medicine.

[b26-arcr-36-1-29] Sen B (2006). The relationship between beer taxes, other alcohol policies, and child homicide deaths. Topics in Economic Analysis & Policy.

[b27-arcr-36-1-29] Senchak M, Leonard KE, Greene BW (1998). Alcohol use among college students as a function of their typical social drinking context. Psychology of Addictive Behaviors.

[b28-arcr-36-1-29] Shiffman S (2009). Ecological momentary assessment (EMA) in studies of substance use. Psychological Assessment.

[b29-arcr-36-1-29] Shiffman S, Stone AA, Hufford MR (2008). Ecological momentary assessment. Annual Review of Clinical Psychology.

[b30-arcr-36-1-29] Storvoll EE, Rossow I, Pape H (2010). Where do adolescents get drunk? A study of the relative importance of various drinking locations among Norwegian adolescents. Nordic Studies on Alcohol and Drugs.

[b31-arcr-36-1-29] Tin ST, Ameratunga S, Robinson E (2008). Drink driving and the patterns and context of drinking among New Zealand adolescents. Acta Paediatrica.

[b32-arcr-36-1-29] Treno AJ, Lee JP (2002). Approaching alcohol problems through local environmental interventions. Alcohol Research & Health.

[b33-arcr-36-1-29] Todd M, Armeli S, Tennen H (2009). Interpersonal problems and negative mood as predictors of within-day time to drinking. Psychology of Addictive Behaviors.

[b34-arcr-36-1-29] Walker S, Waiters E, Grube JW, Chen MJ (2005). Young people driving after drinking and riding with drinking drivers: Drinking locations—What do they tell us?. Traffic Injury Prevention.

[b35-arcr-36-1-29] Wiebe DJ, Blackstone MM, Mollen CJ (2011). Self-reported violence-related outcomes for adolescents within eight weeks of emergency department treatment for assault injury. Journal of Adolescent Health.

[b36-arcr-36-1-29] Wiebe DJ, Guo W, Allison PD (2013). Fears of violence during morning travel to school. Journal of Adolescent Health.

